# Transformative Deep Vein Thrombosis Prophylaxis With Sequential Compression Devices in the Care of Hospitalized Patients

**DOI:** 10.7759/cureus.70639

**Published:** 2024-10-01

**Authors:** Aaisha Shahbaz, Randev A Wannakuwatte, Cara Mohammed, Abdulaziz Alzarooni, Harini Pendem, Farhat Majeed, Venkataramana Kuruba, Sherien Metry, Tanvi Mahajan, Hasim Reza, Mariam Benjamen, Manju Rai

**Affiliations:** 1 Trauma and Orthopedic Surgery, University Hospitals Birmingham, Birmingham, GBR; 2 Surgery, Grodno State Medical University, Grodno, BLR; 3 Orthopedic Surgery, Sangre Grande Hospital, Sangre Grande, TTO; 4 Surgery, Royal College of Surgeons in Ireland, Dublin, IRL; 5 Internal Medicine, Chalmeda Anand Rao Institute of Medical Sciences, Karimnagar, IND; 6 Medicine, Quaid e Azam Medical College, Bahawalpur, PAK; 7 Orthopedics, All India Institute of Medical Sciences Mangalagiri, Mangalagiri, IND; 8 General Practice, Medical University of Assiut, Asyut, EGY; 9 Internal Medicine, Maharishi Markandeshwar Medical College and Hospital, Dinanagar, IND; 10 Medicine, Central America Health Sciences University, Ladyville, BLZ; 11 Medicine, Ain Shams University, Cairo, EGY; 12 Biotechnology, Shri Venkateshwara University, Gajraula, IND

**Keywords:** deep vein thrombosis, dvt prophylaxis, hospitalized patients, patient safety, sequential compression devices, thrombosis prevention, venous thromboembolism

## Abstract

Deep vein thrombosis (DVT) is a critical complication and concern in hospitalized patients due to its significant morbidity and mortality. Given the complex and multifaceted pathophysiology surrounding DVT formation, patients who have had surgical interventions faced acute or chronic trauma and prolonged immobility are at substantially high risk. Identifying these risk factors early is essential for early intervention and prophylaxis. Current standard-of-care prophylaxis for DVT includes pharmacological agents such as anticoagulants, and recently, there has been an increase in the use of mechanical medical devices such as sequential compression devices (SCDs). Pharmacological prophylaxis, while shown to be effective in some patients, carries certain risks of complications such as bleeding. SCDs offer a safer and more effective approach for these patients. SCDs function by artificially replicating the “pumping mechanism” present in the soleus muscle to enhance venous return and reduce stasis. Various types of SCDs, namely intermittent pneumatic compression and graduated compression stockings, have demonstrated clinical efficacy when used as an adjunctive intervention with anticoagulant medications. This narrative review explores the pathophysiology, risk factors, and prophylactic measures for DVT, focusing on the use of SCDs as a non-pharmacological intervention. Through synthesizing evidence from various studies obtained from PubMed, MEDLINE, and Cochrane Library and evaluating the benefits and limitations of SCD use, this review highlights the need for tailored prophylactic strategies, considering patient-specific risk factors and preferences.

## Introduction and background

Deep vein thrombosis (DVT) is a prevalent complication in hospitalized patients that can lead to higher rates of morbidity and mortality if inadequately managed [1]. In clinical settings, DVT stems from elevated venous pressure leading to limb swelling, tenderness, pain, and discoloration of the skin. Additionally, when the thrombosis affects the pelvic and cerebral vessels, this can subsequently result in abdominal pain and severe headaches. The formation of DVTs generally occurs at the lower extremity and pelvis, making these regions essential target areas to prevent complications such as pulmonary embolism (PE) and post-thrombotic syndrome (PTS) [2-4].

Patients who are hospitalized are at an especially high risk of developing DVTs due to prolonged immobility, various pathophysiological conditions, and comorbid risk factors. The Centers for Disease Control and Prevention data from 2007-2009 estimated an average of 348,558 DVT diagnoses annually across all adult hospitalizations in the United States [5]. According to the widely accepted principles of Virchow’s triad, the formation of venous thrombi (VT) is based on three integral factors: blood stasis, vessel damage, and hypercoagulability, with stasis being the most significant contributor [6]. A hypercoagulable state arises when pro-thrombotic factors like tissue factor, factor VIII, and cancer procoagulant outweigh anti-thrombotic factors such as antithrombin III, protein C, and plasmin [7]. Patients with pelvic and lower extremity fractures, major trauma, and those undergoing prolonged ICU treatment are particularly vulnerable to DVT due to extended bed rest, comorbid conditions, and surgical procedures [8-10].

Although DVT is localized, it can eventually lead to PTS and PE when inadequately managed, which can significantly complicate patient outcomes and increase morbidity [11,12]. Thrombi dislodged from the deep veins can travel through the heart and become lodged in the pulmonary vasculature, resulting in PE. Depending on the size and location of the embolism, PE can cause a range of symptoms, from mild chest pain to more drastic consequences such as oxygen desaturation and hemodynamic instability due to increased right ventricular systolic pressure [13]. Injury to the vessels and valves resulting from thrombosis and inflammation induces chronic symptoms in the lower extremities, including edema, neuropathic pain, heaviness, hyperpigmentation, and the development of venous ulcers [14]. Hence, despite its low mortality rate, DVT and the resulting PE significantly diminish the patient’s overall quality of life.

Given the potentially severe consequences of DVT, preventive measures are paramount in its management. Various approaches are utilized for DVT prophylaxis in hospitalized individuals, encompassing anticoagulants, antiplatelet agents, and sequential compression devices (SCDs). SCDs emulate physiological processes like the “muscle pump” mechanism of the soleus, enhancing venous return from the lower limbs and mitigating stasis [15]. SCDs include intermittent pneumatic compression (IPC) devices, compression stockings, foot pumps, and active compression-decompression devices [16,17]. Each of these devices possesses distinct advantages and drawbacks that necessitate thorough deliberation when determining the most suitable option for hospitalized patients. Factors including patient mobility, independence, medical condition, risk profile, and preferences must all be taken into careful consideration.

A significant benefit of systematic compression devices is their low-risk, non-invasive nature, particularly valuable in situations where anticoagulant medications are contraindicated. This review seeks to investigate the effectiveness of SCDs in preventing DVT among hospitalized patients, offering a thorough examination of existing literature and empirical data. This endeavor contributes to the understanding of DVT prevention and promotes patient safety and welfare within hospital settings.

## Review

Methods

A comprehensive literature search was conducted using electronic databases, including PubMed, MEDLINE, and Cochrane Library, to identify relevant studies on DVT and the use of SCDs for its prevention. The search included articles published between January 2000 and December 2023. Keywords used in the search were "deep vein thrombosis," "DVT," "sequential compression devices," "SCD," "prophylaxis," "non-pharmacological intervention," and "venous thromboembolism." Studies were included if they met the following criteria: focused on hospitalized patients at risk for DVT, examined the use of SCDs as prophylaxis for DVT, included data on the efficacy of SCDs in reducing DVT incidence, published in peer-reviewed journals, and available in English. Meanwhile, studies were excluded if they did not deliberately focus on hospitalized patients, were case reports, editorials, or review articles without original data, or lacked patient outcome data on SCD use and efficacy.

Data from the selected studies were extracted and reviewed independently by six authors to ensure accuracy and completeness. The quality of the included studies was evaluated by five authors based on their methodology, including randomization, blinding, and completeness of outcome reporting. This article is based on previously conducted studies and does not contain any data or experiments from new studies with animals or human subjects.

Pathophysiology of DVT

**Figure 1:**
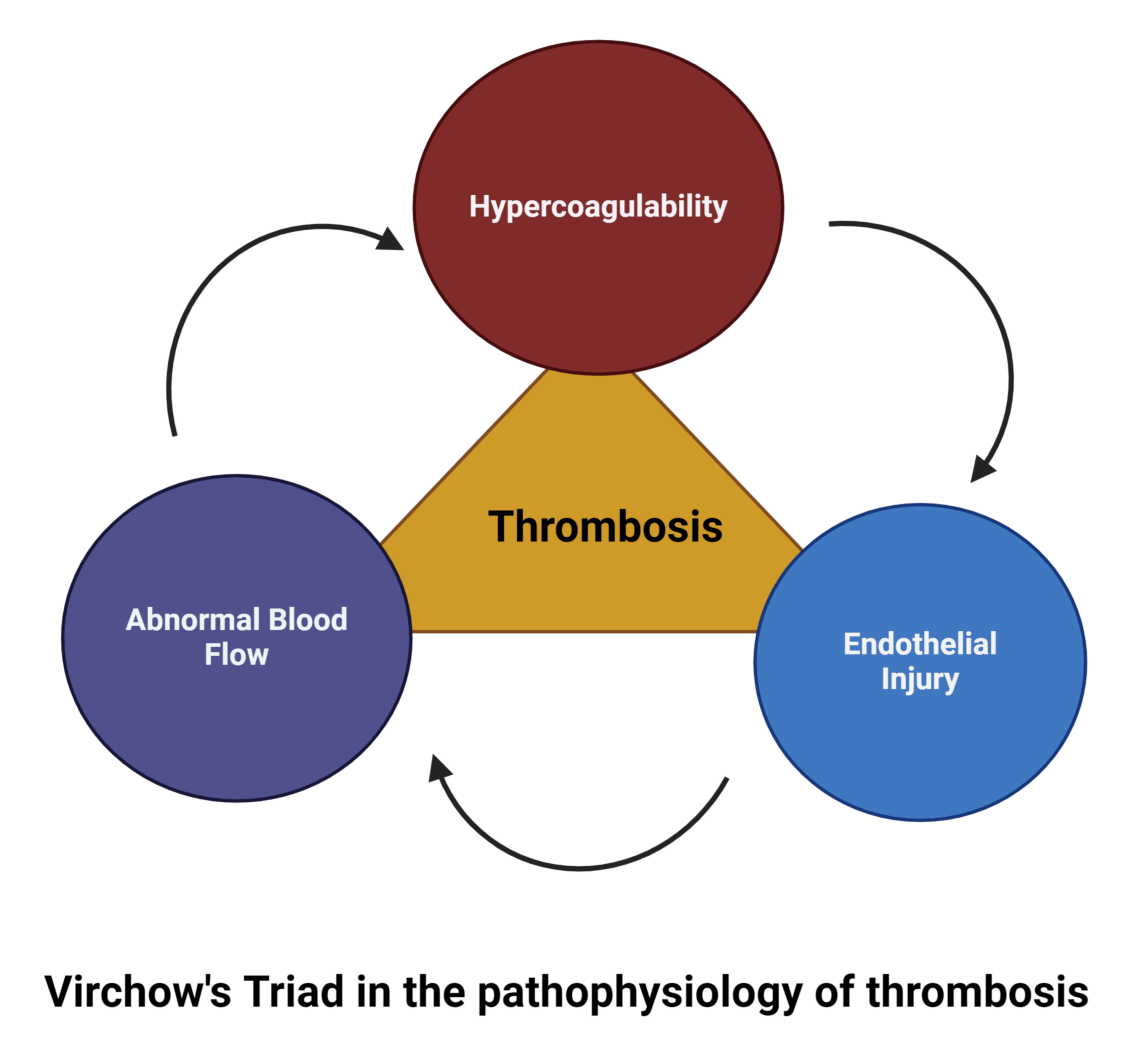
Formation of DVT Formation of DVT is a complex process that is affected by three factors: endothelial damage/vascular injury, blood flow turbulence, and hypercoagulability of blood. (Created with BioRender.com. Licensed for use under the BioRender licensing agreement) DVT: deep vein thrombosis

The formation of DVT is multifactorial and follows a complex process described by Virchow’s triad [6], which includes three main pathophysiological factors that predispose a person to develop vascular thrombosis (Figure 1). These factors include endothelial damage/vascular injury, blood flow turbulence, and hypercoagulability of blood.

An intact endothelium preserves its anticoagulant and anti-platelet characteristics through the secretion of specific specialized cell surface glycoproteins and various molecules, including thrombomodulin, tissue factor, and ectonucleotidases [18]. These components play essential roles in regulating thrombin activity and reducing the prothrombotic impact of nucleotides like adenosine diphosphate [19]. Traumatic injury, surgical procedures, and the placement of intravenous catheters can directly harm the endothelium, inducing a procoagulant condition and activating platelets, thereby initiating thrombus formation [20].

Chronic hypertension and atherosclerotic disease secondary to hyperlipidemia expose subendothelial collagen and tissue factors. This exposure triggers local cytokine release, platelet activation, and leukocyte adhesion to the endothelium, thereby fostering venous thrombosis [18-20]. Inflammation, smoking, and certain medications induce oxidative stress, triggering the release of reactive oxygen species (ROS) and other oxidative agents. These compounds have the potential to compromise endothelial cell function and integrity.

Reduced blood flow, termed blood stasis, plays a pivotal role in blood coagulation and subsequent VT formation. Turbulence, often instigated by arterial wall damage, can occur when blood flows over an impaired surface or at an excessive rate. This can consequently result in disordered flow patterns and eddy currents, which cause a cascade of physiological responses such as heightening flow friction, triggering neutrophil migration beneath the endothelium, provoking p-selectin production, encouraging platelet and leukocyte accumulation, promoting fibrin formation, and prompting the release of endothelial multimeric adhesive glycoproteins [21-23]. Various conditions such as left ventricular (LV) wall akinesis, valvular heart disease, atrial fibrillation, prolonged immobility (e.g., bedridden patients or extended travel), surgery, and trauma may disrupt blood flow dynamics [24,25].

During atrial fibrillation, the atria do not contract effectively, leading to stasis or pooling of blood, particularly in the left atrial appendage. This leads to thrombus formation and subsequent embolic events like stroke and vascular trauma. LV wall akinesis exhibits a similar mechanism [26]. Prolonged immobility, such as bedridden patients or prolonged travel, decreases the normal blood flow in the veins, especially in the lower extremities, leading to blood stasis and subsequent thrombus formation. Chronic venous insufficiency, where the veins struggle to send blood back to the heart, can result in blood pooling in the legs. Surgery or trauma can directly disrupt the blood flow dynamics owing to prolonged immobilization and endothelial injury [6].

Hypercoagulability refers to an increased tendency for blood to clot in the absence of bleeding due to an imbalance between pro-thrombotic and anti-thrombotic factors [6]. A deficiency in anticoagulants or overactivity of procoagulant factors can lead to a hypercoagulable state and subsequent thromboembolism [6,26].

Conditions that can lead to hypercoagulable states include antithrombin III deficiency, protein C and S deficiency, hyperhomocysteinemia, elevated factor VIII, antiphospholipid syndrome, malignancy, smoking, endogenous and exogenous hormones, and trauma [7,27].

Risk factors for DVT

**Figure 2:**
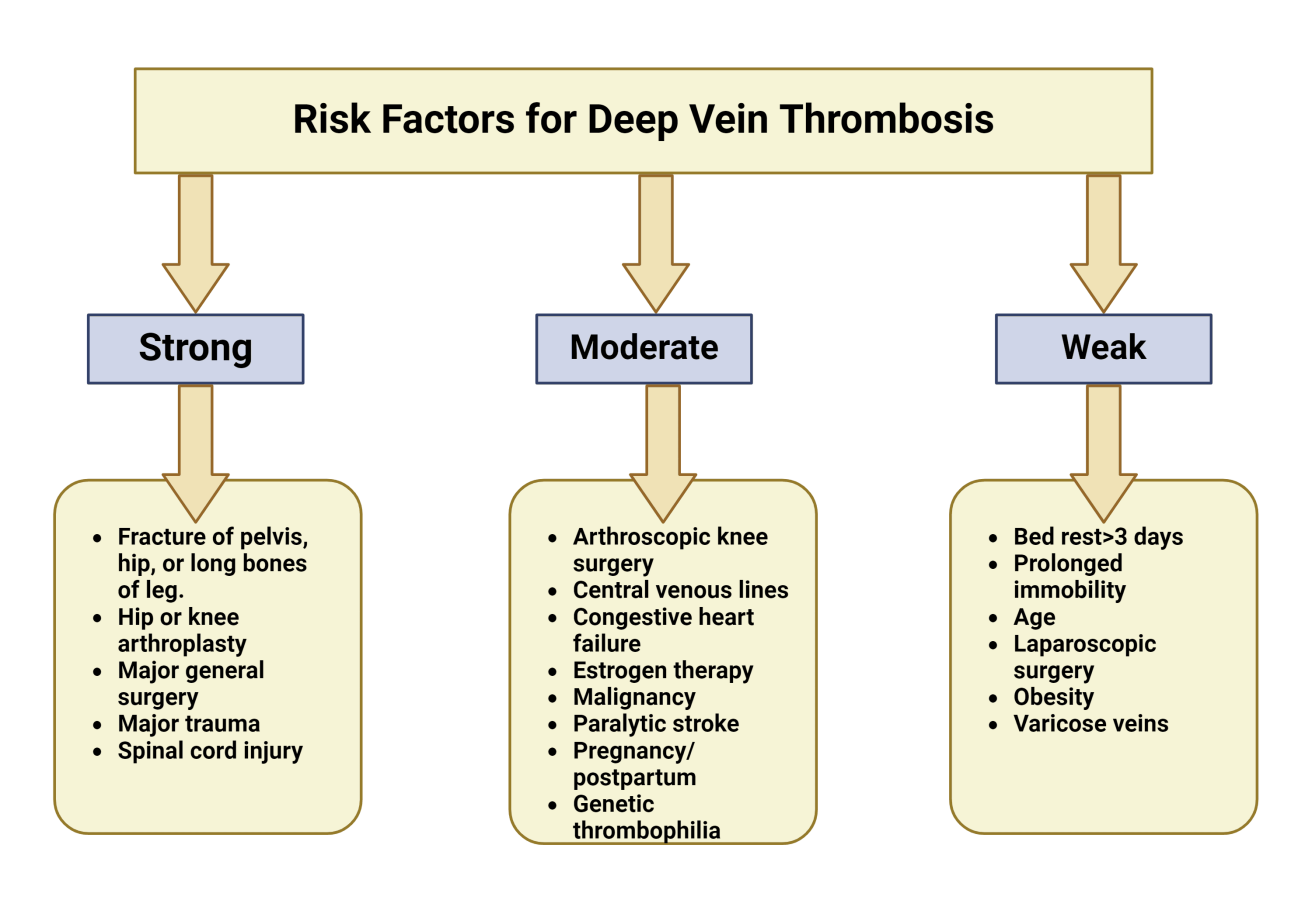
Risk factors of DVT Various non-modifiable and modifiable risk factors may contribute to DVT development and prognosis. Some of the factors that are of weaker correlation include patient immobility (or bed rest) greater than three days, patient age, history of laparoscopic surgery, obesity, and presence of varicose veins. Some of the factors that have a moderate correlation include a history of arthroscopic knee surgery, placement of central lines, history of congestive heart failure, estrogen therapy, paralytic stroke, pregnancy/postpartum, and genetic thrombophilia. Some of the strongest predictors of DVT are comorbid fractures of the pelvis, hip or long bones of the lower extremity, hip or knee arthroplasty, major general surgery, major trauma, and spinal cord injury. (Created with BioRender.com. Licensed for use under the BioRender licensing agreement)

Identifying and establishing the risk factors associated with DVT is crucial, as these factors are prevalent and standard across most hospitalized DVT patients [28]. Nonmodifiable patient-related risk factors include genetic predisposition, sex, and race. Modifiable risk factors encompass aspects such as BMI, lifestyle choices, and smoking (Figure 2). Recognizing these risk factors is essential for identifying high-risk patients and initiating early prophylactic measures.

Genetic risk factors contribute to the majority of thrombophilic disorders. Some predisposing genetic conditions include hypercholesterolemia, hyperhomocysteinemia, prothrombin gene mutation, factor XII deficiency, protein C and S deficiencies, and having a non-O blood type [29]. Certain races, such as African-Americans, have a higher risk of developing DVT compared to Caucasians [30].

Moreover, individuals who are hospitalized with infectious conditions such as COVID-19, tuberculosis, or sepsis are at a significantly increased risk of developing DVT [31]. Chronic conditions such as chronic kidney disease, diabetes mellitus, nephrotic syndrome, and hyperhomocysteinemia, along with some autoimmune illnesses, such as lupus, rheumatoid arthritis, and vasculitis, can lead to a hypercoagulable state, which increases the risk of developing DVT [7,32,33].

There is a significant likelihood of recurrent thromboembolism, especially in hospitalized persons with other risk factors. Recurrent DVT is linked to a history of DVT and a history of PE [34]. Additionally, certain medications such as hormone replacement therapy, contraceptives, antidepressants, tamoxifen, testosterone, heparin, and glucocorticoids elevate the likelihood of developing DVT due to the potential of local harm to deep veins [35].

**Figure 3:**
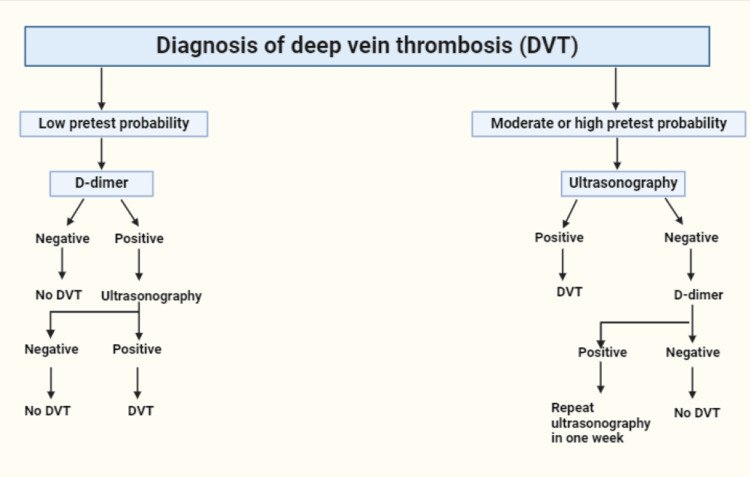
Diagnostic Tools to Screen for DVT There are two predominant diagnostic tools to screen for DVT. The D-dimer test offers low pretest probability and is recommended to rule out DVT in low-risk patients. The Doppler ultrasound is recommended for high-risk patients and offers high pretest probability. (Created with BioRender.com. Licensed for use under the BioRender licensing agreement) DVT: deep vein thrombosis

The Wells criteria (WC) is a diagnostic tool utilized to evaluate a patient's susceptibility to DVT by assigning scores to different risk variables [36]. These variables include active cancer, recent major surgery, prolonged bed rest, diffuse leg or calf swelling, localized deep vein tenderness, lower limb paralysis, pitting edema in the symptomatic leg, and a past history of DVT. Based on the total score on the WC, patients are categorized into low risk (5%), moderate risk (17%), or high risk (17-53%) (Figure 3). For low- to moderate-risk patients, a D-dimer test is recommended to rule out DVT [37]. For high-risk patients, a Doppler ultrasound is advised for a finalized diagnosis [38]. The risk factors for DVT are weighed to ensure accurate diagnosis, initiate prophylaxis, and start immediate treatment.

DVT prophylaxis

DVT prophylaxis treatment primarily aims to inhibit and prevent thrombus propagation, recurrent thromboembolic events, acute PE, and the incidence of long-term sequelae such as chronic pulmonary hypertension and PTS [39]. Prophylactic modalities for DVT can be pharmacological and/or device assisted.

Pharmacological Prophylaxis

Although the 2011 American Academy of Orthopedic Surgeons guidelines recommend the use of mechanical prophylaxis, pharmacological options have also shown efficacy in the prevention of DVT [40]. However, each pharmacological agent has specific indications, mechanisms of action, routes of administration, and limitations. The selection of an appropriate pharmacological agent requires a comprehensive evaluation of the patient’s underlying comorbidities, bleeding risk, and concomitant medication history to optimize safety and efficacy. For example, anticoagulants such as low-molecular-weight heparin (LMWH), unfractionated heparin (UFH), or vitamin K antagonists are normally considered for patients with a higher risk of DVT formation [10]. These medications are often initiated before surgery and continued for a specified duration postoperatively or during periods of immobility.

Several studies have demonstrated the efficacy of low-dose UFH in reducing the risk of DVTs [41]. In addition, while the use of the antiplatelet medication aspirin may be considered controversial [10], aspirin-based thromboprophylaxis was found to be as effective as LMWH in preventing mortality in patients experiencing lower extremity fractures managed surgically. Specifically, aspirin use was associated with low occurrences of DVT and PE, as well as a low 90-day mortality rate [42]. Comparative characteristics of LMWH, direct oral anticoagulant (DOAC), and warfarin are described in Table 1. However, some concerns have also been reported with the use of pharmacological agents. For example, pharmacological interventions modulate the blood coagulation profile, posing a significant drawback due to the potential risk of bleeding, especially in surgical contexts. These alterations may predispose individuals to joint hematomas, post-joint replacement procedures, and intracranial hemorrhage following head trauma or neurosurgical interventions [43].

**Table 1:** Comparative characteristics of LMWH, DOAC, and warfarin LMWH: low-molecular-weight heparin, DOAC: direct oral anticoagulant, FDA: Food and Drug Administration, INR: international normalized ratio

Characteristics	LMWH	DOAC	Warfarin
Mechanism of action	Enhances activity of antithrombin, inhibiting factor Xa and IIa	Apixaban, edoxaban, and rivaroxaban are direct and reversible inhibitors of factor Xa; dabigatran is a reversible inhibitor of free thrombin, fibrin-bound thrombin, and thrombin-induced platelet aggregation	Inhibits vitamin K-dependent clotting factors (II, VII, IX, X) and anticoagulant proteins C and S
Route of administration	Subcutaneous injection	Oral	Oral
Onset of action	Rapid (within hours)	Rapid (within hours)	Slow (2-3 days)
Monitoring	Not required to monitor INR for routine use; regular follow-up recommended	Not required to monitor INR for routine use; regular follow-up recommended	Regular INR monitoring for warfarin
Reversal agents	Protamine sulfate	Specific reversal agents (e.g., idarucizumab for dabigatran, andexanet alfa for factor Xa inhibitors)	Vitamin K, fresh frozen plasma
Risk of bleeding	Moderate	Low to moderate	High
FDA approved agents	Enoxaparin, dalteparin	Rivaroxaban, apixaban, dabigatran	Warfarin

Non-pharmacological Prophylaxis

Despite the efficacy of pharmacological prophylaxis for DVT prevention, there are still uncertainties regarding the optimal agent, dose rate, and contraindications [44]. Mechanical, or non-pharmacological prophylaxis is considered a standard approach for DVT, along with anticoagulant therapy. SCDs, which are specialized mechanical devices, are used independently or as an adjunct with pharmacological options for venous thromboprophylaxis. Although the precise physiological mechanism of action of SCDs remains uncertain, it is hypothesized that the therapeutic benefit arises from the compressive sleeve of the device which alleviates stasis and hypercoagulability [16]. A typical setup involves placing a sleeve around the lower limb and linking it to a pump. This sleeve is designed with chambers that are sequentially inflated and deflated.

The process usually begins at the ankle and progresses up to the knee. This ensures a systematic compression along the limb with each pump filling each chamber with air up to a predetermined pressure level. Once inflated, the air is maintained for a specific time duration before being released. Following the deflation of the sleeves, a designated rest interval ensues before the sequential compression cycle recommences. This intermittent inflation and deflation are thought to mimic walking, thus enhancing venous return and relieving stasis [16]. This also improves endothelial function by promoting the release of tissue plasminogen activator, which helps break down clots.

**Figure 4:**
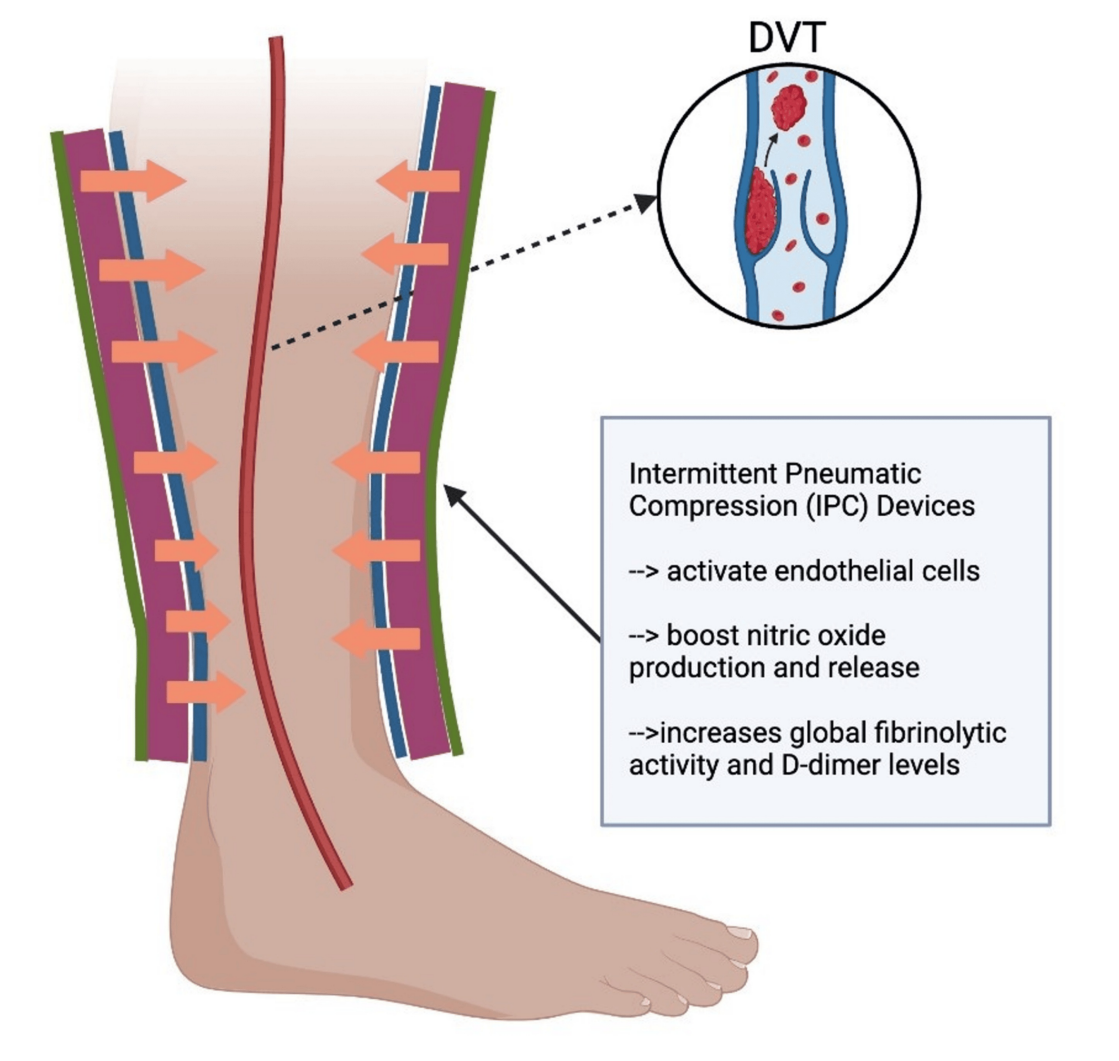
Mechanism of action of IPC devices IPC devices are types of SCDs that function through activation of endothelial cells. This leads to nitric oxide production and release, an increase in global fibrinolytic activity, and D-dimer levels. IPCs have demonstrated efficacy in aiding thrombosis prevention and vascular homeostasis maintenance and are a commonly recommended intervention for DVT prophylaxis in surgical patients. The orange arrows pointing inward toward the leg indicate the compression provided by the IPC device (which is indicated by the blue, purple, and green lines covering the outside of the leg). The dashed black arrow indicates a DVT forming in the vessel (indicated by the red line inside the leg). (Created with BioRender.com. Licensed for use under the BioRender licensing agreement) IPC: intermittent pneumatic compression, SCDs: sequential compression devices, DVT: deep vein thrombosis

IPC devices, which are a subtype of SCDs, activate endothelial cells. This subsequently boosts nitric oxide production and release, which is a critical component of Virchow's triad that aids in thrombosis prevention and vascular homeostasis maintenance (Figure 4). Additionally, IPC increases global fibrinolytic activity and D-dimer levels, with evidence suggesting a rise in prostacyclin levels [16]. IPC and graduated compression stockings (GCS) are common mechanical prophylaxis and are commonly recommended for DVT prophylaxis in surgical patients [45]. Both IPC and GCS have distinct practical properties and implications. IPC works by cyclically applying pressure to the deep veins of the limb, thereby actively pushing blood towards the heart and reducing stasis. On the other hand, GCS exerts continuous pressure on the limb, which helps keep the veins narrower and prevents blood from pooling [45].

Effectiveness and utility of SCDs

Different SCDs can be used in combination with each other or with pharmacological agents. Their combined use has demonstrated synergistic effects in some studies. For example, Vignon et al. reported that the concurrent use of IPC and GCS yields a more pronounced reduction in the occurrence of VTE compared to each therapy alone [46]. Similarly, various systematic reviews have consistently reported enhanced outcomes when IPC is combined with pharmacological prophylaxis [47,48]. These reviews have further indicated that IPC can effectively decrease the occurrence of VTE in surgical patients. Despite these benefits, adherence to non-pharmacological approaches remains a concern. Greenall and Davis described several factors that impact compliance with IPC, including patient discomfort, mobilization, equipment supply and demand, and healthcare professional knowledge and behaviors [49]. Furthermore, although SCDs have the advantage of being non-invasive, their high initial cost can be a significant limitation, particularly in resource-limited healthcare environments. Apart from cost concerns, other drawbacks include potential fitting and sizing issues and skin irritation.

Comparative studies

Several studies have investigated the effectiveness of SCDs as mechanical prophylaxis in preventing DVTs in hospitalized patients. A study by Dennis et al. showed that the primary outcome, the occurrence of DVT, was observed in 8.5% of patients in the IPC-using group compared to 12.1% in the no IPC-using group, resulting in an absolute risk reduction of 3.6% and a relative risk reduction of 0.69 [50]. Secondary outcomes such as mortality post-treatment, skin breaks, and falls with injury were also examined. IPC was associated with a lower incidence of skin breaks but showed no significant difference in mortality or falls with injury. Additionally, IPC treatment was linked to improved survival during the six-month follow-up, but there were no changes in disability outcomes [50].

Similarly, a study by Sachdeva et al., predominantly focusing on general and orthopedic surgery patients, showed that the incidence of DVT was significantly lower in the treatment group using GCS compared to the control group without GCS use [51]. This study revealed that the incidence of proximal DVT and PE was also reduced in the GCS group. However, data on adverse effects were limited. The authors concluded that GCS effectively reduces the risk of DVT in hospitalized patients, particularly in surgical settings, although evidence for their efficacy in medical patients is sparse [51]. Furthermore, a study performed by Dennis et al. revealed that DVT formation in the popliteal or femoral veins detected on Doppler ultrasound occurred in 10.0% of patients in the “thigh-length GCS” group and in 10.5% of patients in the “non-GCS” group, resulting in a non-significant absolute risk reduction rate of 0.5% [50]. These findings suggest that thigh-length GCS do not appear to reduce the risk of DVT after stroke, questioning the need for its use in this patient population and indicating a potential need for revision of national stroke guidelines.

A prospective cohort study by Arabi et al. investigated the impact of mechanical thromboprophylaxis with IPC and GCS on the incidence of VTE and hospital mortality among critically ill medical-surgical patients in the ICU setting. The results revealed that incident VTE occurred in 7.1% of cases, and the use of IPC was associated with a significantly lower incidence of VTE compared to no mechanical thromboprophylaxis, with a propensity score-adjusted hazard ratio of 0.45 [52]. The benefits of IPC use were not impacted by the type of prophylactic heparin used, the incidence of recent trauma, or surgical admission. GSC, on the other hand, was not associated with a significant reduction in VTE. Similarly, several studies also assessed the efficacy of SCDs when used in combination with or in comparison with pharmacological prophylaxis. Maradei-Pereira et al. investigated the effects of a unilateral portable IPC device compared to enoxaparin on postoperative edema and blood loss [53]. Their randomized trial included patients undergoing total knee arthroplasty (TKA). The findings showed that IPC was associated with less swelling and blood loss compared to enoxaparin.

Additionally, Kamachi et al. compared IPC with enoxaparin to IPC alone in a 1:1 ratio after laparoscopic surgery for gastric and colorectal malignancies [54]. The primary outcome, VTE occurrence evaluated by multidetector CT on day 7, showed no significant differences between groups. However, proximal DVT and/or PE were less frequent in the IPC with concurrent enoxaparin use group. While there were bleeding events reported in the IPC with the enoxaparin group, these events were quickly managed by discontinuation of the drug. Overall, IPC with enoxaparin did not reduce the rate of VTE in this patient population. In another study, Lobastov et al. included patients undergoing major surgery with a Caprini score of ≥11 who received IPC with standard prophylaxis or standard prophylaxis alone [55]. Their findings showed that patients receiving IPC in addition to standard prophylaxis had a significantly lower incidence of asymptomatic VT. PE occurred in none of the IPC groups and in 2.5% of the control group, while postoperative death rates were 2.9% in the IPC group and 4.9% in the control group. This study highlights that adjunctive IPC appears to be beneficial in reducing the risk of VT in high-risk surgical patients.

Moreover, a study done by Shalhoub et al. highlighted that VTE occurred in 1.7% of patients in the LMWH-only group and 1.4% in the LMWH and GCS group, with a risk difference of 0.30% [56]. As the confidence interval did not cross the non-inferiority margin of 3.5%, LMWH alone treatment was confirmed to be non-inferior. These findings suggest that for patients undergoing elective surgery and at moderate or high risk of VTE, pharmacological thromboprophylaxis alone is as effective as a combination of pharmacological thromboprophylaxis and GCS, indicating that GCS may be unnecessary in most cases. Table 2 describes the characteristics of the studies included in the non-pharmacological management of DVT.

**Table 2:** Summary of studies that assessed the efficacy of SCDs (IPCs and GCSs) for DVT prophylaxis RCT: randomized controlled trial, IPC: intermittent pneumatic compression, UK: United Kingdom, DVT: deep vein thrombosis, GCS: graduated compression stocking, TKA: total knee arthroplasty, VT: venous thrombosis, LMWH: low-molecular-weight heparin

Author	Study type	Participants	Participants’ characteristics	Setting	Intervention	Outcomes	Findings
Dennis et al. (2015) [50]	RCT	2876; 1438 IPC, 1438 no IPC	Acute stroke and were immobile	94 UK hospitals	Routine care or routine care + IPC	DVT in popliteal or femoral veins; any symptomatic DVT in the proximal veins	DVT in 8.5% of the IPC group and 12.1% in the group without IPC
Sachdeva et al. (2018) [51]	Review	1681	Conditions other than stroke	20 RCTs	GCS, no GCS	DVT	DVT in 9% in the GCS group compared to 21% in the non-GCS group
Dennis et al. (2009) [57]	RCT	2518; GCS (n=1256) or no GCS (n=1262)	Acute stroke and were immobile	64 centers in 3 countries	Routine care or routine care + thigh-length GCS	Symptomatic or asymptomatic DVT in the popliteal or femoral veins	DVT in 10% of the GCS group and 10.5% in routine care
Arabi et al. (2013) [52]	Prospective cohort study	798, IPC (n=229), GCS (n=180), no mechanical thromboprophylaxis (n=389)	Surgical patients	ICU	IPC, GCS, no mechanical thromboprophylaxis	VTE	Incidence of VTE: 4.8% in IPC, 10% in GCS, and 7.2% in no mechanical thromboprophylaxis
Maradei-Pereira et al. (2022) [53]	RCT	150; mechanical (n=75), enoxaparin (n=75)	TKA	Hospital	IPC, enoxaparin	Edema, blood loss	No VTE case in both groups; edema: increase in leg circumference was 2 cm in the enoxaparin group compared to 1.5 in IPC (p<0.001); loss of blood: 566.1 ml in enoxaparin vs 420.8 ml in IPC
Kamachi et al. (2020) [54]	RCT	448; 208 in IPC group, 182 in IPC with enoxaparin	Surgical patients	15 hospitals	IPC and IPC + enoxaparin	VTE	VTE in 4.8% in IPC compared to 3.8% in IPC + enoxaparin
Lobastov et al. (2021) [55]	RCT	407; 204 in the IPC group, 203 in the control group	Surgical patients	2 institutes	IPC + low-molecular-weight heparin, standard prophylaxis	VT	VT in 0.5% in the IPC group compared to 16.7% in the control group
Shalhoub et al. (2017) [56]	RCT	1888; 948 LMWH, 940 LMWH + GCS	Surgical patients	7 hospitals	LMWH, LMWH + GCS	Lower limb DVT	DVT in 1.7% in LMWH and 1.4% in LMWH + GCS

Barriers and future guidelines in the implementation of SCDs

SCD adoption in a hospital setting encounters various obstacles, such as patient adherence, cost efficiency, and resource availability. These parameters are essential in determining the effectiveness of SCDs in preventing DVT [57-59].

Patient compliance poses significant challenges to the effective implementation of SCDs. Dennis et al. and Kim et al. demonstrate that even when SCDs are easily accessible, the rates at which they are actually used are often below ideal [52,58]. Obi et al. found that adherence to standard SCDs was at 47%.59 The primary concerns mentioned were patient mobility and ease of usage. Kim et al. further emphasized that patient pain and sleep disruption negatively affected compliance, suggesting that despite the availability of the treatment, inconsistent utilization was caused by discomfort and a lack of follow-up from healthcare practitioners [58].

Reduced utilization of SCDs can be attributed to economic considerations such as the high cost of devices and the requirement for continuous maintenance and replacement of components. Obi et al. examined economic issues related to the availability of resources and how they impacted the compliance and effectiveness of SCDs [59]. The expense associated with SCDs and their upkeep presents a substantial obstacle to their extensive implementation. Ritsema et al. highlighted that a significant obstacle to SCD effectiveness is their limited accessibility at the point of treatment [60]. Hospitals often face the challenge of insufficient or unavailable medical devices. The situation is especially noticeable in large hospitals and institutions with a high incidence of patient turnover. A study conducted by Braithwaite et al. confirmed this, demonstrating that the availability and implementation of SCDs varied significantly based on the geographical regions and types of hospitals [61]. Cost-benefit assessments indicate that despite the high initial and ongoing costs of SCDs, their effective utilization can lead to a reduction in long-term healthcare spending by reducing complications related to DVT [62].

To address these challenges, additional investigation into the utility of SCD for the prevention of DVT should prioritize various elements. Firstly, further research to investigate the impact of educational initiatives or identify the psychological obstacles to adherence to SCD is warranted. Chen et al. found that while provider education led to a considerable increase in the frequency of SCD orders, it did not result in a corresponding rise in patient compliance rates [63]. This discovery highlights the need for educational initiatives that actively involve patients in order to tackle individualistic obstacles to compliance. Research has indicated that incorporating wearable technologies and the Internet of Things for ongoing monitoring and feedback could potentially enhance compliance [63].

Technological advancements have the potential to affect the utilization and effectiveness of SCDs. According to Palmerola et al., the introduction of battery-powered or cordless SCDs could potentially resolve problems related to mobility and comfort. Incorporating sensors that monitor and actively promote adherence in future device designs may further help enhance compliance [64].

Therefore, it is crucial to conduct additional research and allow for technological breakthroughs to overcome the obstacles of compliance, cost, and resource availability. This will ultimately lead to enhanced patient outcomes [63,52,60,64]. By integrating cutting-edge technologies and extensive patient education programs, healthcare practitioners can improve the efficacy of SCDs in clinical practice.

## Conclusions

Given the significant risk presented by DVT in hospitalized patients, effective management and prophylaxis are crucial to mitigate severe adverse events such as PE and post-thrombotic syndrome. SCDs, in particular, offer a non-invasive alternative or adjunct to anticoagulant therapy, and numerous studies have underscored the efficacy of these innovative devices in reducing DVT incidence, though challenges in adherence and cost persist. Future research and clinical guidelines should aim to optimize the integration of mechanical and pharmacological prophylaxis, considering patient-specific factors and resource availability. Personalized risk assessment tools, such as the WC, can guide targeted interventions, ensuring timely and effective DVT prevention. A comprehensive approach that combines evidence-based practices with innovative solutions can be ultimately utilized to improve hospitalized patients’ care, safety, and outcomes.
